# Rheological Behavior of Amino-Functionalized Multi-Walled Carbon Nanotube/Polyacrylonitrile Concentrated Solutions and Crystal Structure of Composite Fibers

**DOI:** 10.3390/polym10020186

**Published:** 2018-02-14

**Authors:** Hailong Zhang, Ling Quan, Fengjun Shi, Changqing Li, Huanqiang Liu, Lianghua Xu

**Affiliations:** 1 School of Civil Engineering and Communication, North China University of Water Resources and Electric Power, Zhengzhou 450045, China; shifengjun1962@126.com (F.S.); liuhuanqiang@ncwu.edu.cn (H.L.); 2 Key Laboratory of Carbon Fiber and Functional Polymers Ministry of Education, Beijing University of Chemical Technology, Beijing 100029, China; licq@mail.buct.edu.cn (C.L.); xulh@mail.buct.edu.cn (L.X.); 3 School of Electric Power, North China University of Water Resources and Electric Power, Zhengzhou 450045, China; quanling@ncwu.edu.cn

**Keywords:** carbon nanotubes, polyacrylonitrile concentrated solutions, rheology, X-ray diffraction, mechanical properties

## Abstract

The rheological behavior of amino-functionalized multi-walled carbon nanotubes (amino-CNTs)/polyacrylonitrile (PAN) concentrated solutions in the dimethyl sulphoxide solvent and the effects of the amino-CNTs on the PAN precursor fibers by wet-spinning method were investigated. The amino-CNT/PAN concentrated solutions prepared by in situ solution polymerization with homogeneous dispersion of amino-CNTs have higher complex viscosity, storage modulus and loss modulus as compared to the control PAN concentrated solutions containing 22% PAN polymer by mass. The composite fibers with amino-CNTs of 1 wt % have lower degree of crystallization, crystal size and crystal region orientation compared to the control PAN precursor fibers. However, the amino-CNT/PAN composite fibers with diameter of about 10.5 μm exhibit higher mechanical properties than the control PAN precursor fibers with diameter of about 8.0 μm. Differential scanning calorimetry analysis demonstrated that the cyclization reaction in composite fibers have broad exothermic temperature range and low exothermic rate. These results indicate that the addition of amino-CNTs into PAN precursor fibers is beneficial to controlling the process of thermal stabilization and obtaining the higher performance of composite fibers.

## 1. Introduction

Carbon nanotubes (CNTs), one-dimensional carbon allotrope used as filler, attracted many researchers owing to its remarkable structure and excellent properties [1,2,3,4], which were considered as an ideal reinforcement for the fabrication of CNT/polymer composites. However, CNTs are easily aggregated and difficult to dispersed in the polymer matrix owing to their strong intermolecular van der Waals interactions and high aspect ratio. Moreover, the weak interfacial interaction between CNTs and the polymer matrix effectively limits the load transfer from the matrix to the CNTs. So, uniform dispersion of CNTs in the polymer matrix and strong interfacial interaction between them are the main challenges for the CNT/polymer composite, which can be used in the technological sectors and branches of industry depending on the CNTs unique properties [5].

Currently, chemical modification on the surface of CNTs could enhance the dispersion and interfacial interaction between the CNTs and polymer matrix. Gulotty et al. [6] reported that the carbon nanotubes with carboxylic functionalization improved their dispersion properties in the polymer nanocomposites, and the coupling interaction between them. Szatkowski et al. [7] reported that the functionalized carbon nanotubes with hydroxyl groups (–OH) were homogeneously dispersed in the polyurethane (PU) matrix and the strong interaction was existed between the PU structure and –OH groups on the functionalized CNTs. However, the covalent functionalization is a double-edged sword because it can reduce aggregation and facilitate CNT interfacing with other materials, but it can also have detrimental effects on CNT properties if not controlled [8,9]. Furthermore, in situ polymerization is considered as the appropriate method for producing the CNT/polymer composites with excellent properties by means of ultrasonication [10,11]. Saeed et al. [12] reported that the functionalized multi-walled carbon nanotubes with aromatic amine (COC_6_H_4_–NH_2_) groups into nylon-6 matrix have homogeneous dispersion by in situ polymerization and enhanced the specific strength and modulus compared to the neat nylon-6 nanofibers. Zhang et al. [13] obtained the uniform dispersion of functionalized CNTs containing hydroxyl groups in the polyacrylonitrile (PAN) matrix and strong interfacial interaction between them through aqueous deposition polymerization. Ultrasonication as an auxiliary method is used to improve the dispersion of CNTs in the polymer matrix. However, the long ultrasonication process and the high power would shorten the length of CNTs [14,15]. Therefore, the ultrasonic parameters play an important role in utilizing the excellent properties of CNTs in composite materials.

PAN is the main precursor for manufacturing carbon fiber with excellent mechanical properties, and belongs to a semi-crystalline polymer which could change the crystal structure during processing of the PAN precursor fiber [16]. After stabilization and carbonization, PAN precursor fiber finally becomes the carbon fiber with outstanding tensile strength, which is widely used in sports, aerospace, automobile, industry and so on. The addition of CNTs into PAN polymer could change the crystal structure [17,18], and the functionalized groups on the surface of CNTs also influence the interfacial interaction between CNTs and PAN polymer [19]. Newcomb et al. [20] obtained the PAN/CNT composite fibers by gel spun and regarded that the addition of CNTs into PAN matrix increased the crystallinity, crystal size and mechanical properties compared to the PAN fibers. He groups [21] reported that PAN/functionalized CNTs with carboxyl acid groups (–COOH) prepared by plasticized spinning exhibited excellent tensile strength and modulus compared to the precursor fibers, and the CNTs–COOH initiated the crystallization and improved the crystal size. Quan et al. [22] prepared the CNT/PAN nascent composite fiber by wet-spinning and regarded that the addition of CNT into PAN nascent fibers increased the degree of crystallinity, crystal size and crystal orientation compared to the control PAN nascent fibers, and the composite fibers had higher tensile strength and tensile modulus than the PAN fibers.

However, few studies are reported on rheological properties of functionalized CNT/PAN concentrated solutions by in situ polymerization. Rheological properties of the solutions can reflect the internal structure and processability, which can provide information about the network structure, dispersion state of the CNTs, and the interfacial interaction between CNTs and polymers [23,24,25,26]. At low frequency, the rheological behaviors reflect the relaxation and motion of polymer chains. At high frequency, the rheological behaviors are corresponding to the movement of polymer chains within small time. So, rheological behaviors of various CNT/polymer composite have been widely studied [27,28,29,30,31]. Kim et al. [32] reported that the addition of amino-CNTs into poly(methyl methacrylate) (PMMA) composite increased the viscosity, storage modulus and loss modulus compared with the control PMMA polymer, which were ascribed to the strong interaction between amino-CNTs and PMMA matrix.

In this paper, we studied the effect of amino-functionalized multi-wall carbon nanotubes (amino-CNTs) on the rheological behavior of PAN/dimethyl sulfoxide (DMSO) concentrated solutions by in situ polymerization and the crystal structure of PAN precursor fibers by wet-spinning method. The effects of amino-CNTs on the rheological behaviors were examined by the parallel plate rheometer. The dispersion of amino-CNT into PAN concentrated solutions and morphology of composite fibers were observed by transmission electron microscopy (TEM) and scanning electron microscopy (SEM), respectively. The interfacial interaction between amino-CNTs and PAN polymer in composite fibers was characterized by the Raman spectroscopy and Fourier transform infrared (FTIR) spectrum. The degree of crystallization, crystal size and crystal region orientation of amino-CNT/PAN composite fibers were measured by the different X-ray diffraction (XRD), respectively. The cyclization reaction was tested in the differential scanning calorimetry (DSC) in nitrogen atmosphere. The mechanical properties were measured by monofilament tensile testing machine.

## 2. Experimental

### 2.1. Materials

Multi-wall carbon nanotubes (CNTs) produced by the chemical vapor deposition method were purchased from Shenzhen Nanotech Port. Co. Ltd. (Shenzhen, China), which diameter and length is about 10–15 nm and 1–10 μm according to the supplier’s specification, respectively. Amino-CNTs were obtained by a series of chemical methods, which could be seen in our previous publication [33,34]. The pristine CNTs firstly need to be purified to remove the metallic catalysts, and then were modified through the 30% H_2_O_2_ solution to obtain the functionalized CNTs with carboxylic acid. The amino-CNTs were obtained after acyl chlorination reaction and amination with triethylenetetramine. The amount of amino groups on the surface of CNTs calculated from the Thermogravimetric curves was 4.87% [34]. Acrylonitrile (AN) monomer with purity >98% was purchased from the Beijing Xingjin Chemical Factory (Beijing, China) and purified by distillation to remove inhibitors before polymerization. 2,2′-Azobisisobutyronitrile (AIBN) was purchased from Beijing Chemical Reagents Company (Beijing, China) and purified by recrystallization. Itaconic acid (IA) and DMSO were purchased from the Sinopharm Chemical Reagent Company Limited (Shanghai, China), and all other reagents used in this paper were the analytical grade without further purification.

### 2.2. In Situ Polymerization of Amino-CNT/PAN Concentrated Solutions

Amino-CNTs were dispersed into the DMSO solvent with the ultrasonication at 60 °C for 1 h. The applied power of ultrasonication bath is 250 W without adjustment, and the ultrasonic frequency is 40 KHz. Then the AN and IA monomer with the molar ratio 99:1 were added into above mixture solutions for 0.5 h. In situ polymerization is initiated by adding the AIBN under mechanical agitation without ultrasonication. After 48 h, the amino-CNT/PAN concentrated solutions were obtained. The concentration of PAN in composite solutions is about 22%, and the mass ratio of amino-CNTs to PAN is about 1%. The control PAN concentrated solutions were prepared at the same conditions without amino-CNTs, which concentration is about 22%. 

### 2.3. Wet-Spinning of Amino-CNT/PAN Composite Fibers

**Figure 1:**
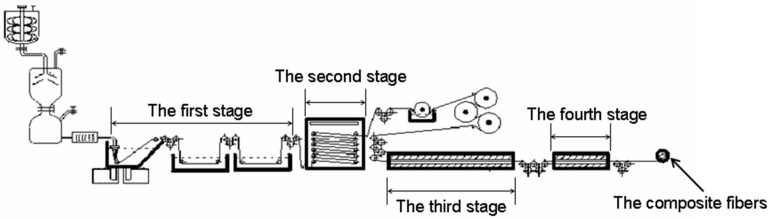
The schematic diagram for wet-spinning method.

The amino-CNT/PAN composite fibers were obtained by wet-spinning method from the concentrated solutions after four-stage stretching. The concentrated solutions were spun into the coagulation bath with the mixture of DMSO and de-ionized water at room temperature to obtain the nascent fibers. After three-stage stretching into the coagulation bath, the first-stage fibers were obtained. The second-stage stretching fibers were performed by passing the fiber through water washing equipment to remove the DMSO solvent under stretching. Then the fibers were stretched under high temperature to obtain the three-stage fibers. Finally, the four-stage stretching fibers were obtained under heat setting. The schematic route is shown in Figure 1. As compared to the composite fibers, the control PAN fibers were prepared at the same conditions from the PAN concentrated solutions.

### 2.4. Characterization

#### 2.4.1. Rheology

Rheological measurements were carried out using a parallel plate rheometer with parallel plate geometry (RS150 Rheostress, Hakke, Germany). In steady-state tests, the curve of flow was determined with a cone-plate sensor (C35/2° Ti) and a gap size of 0.104 mm. The shear rate ranged from 0.3163 s^−1^ to 1000 s^−1^ at different temperatures (40, 50, 60 °C). In dynamic regime, the curve of flow was obtained by increasing the shear rate imposed on the samples from 1.748 s^−1^ to 373.5 s^−1^ with a cone-plate sensor (C20/1° Ti) and a gap size of 0.048 mm. The measurements were done at a constant shear stress of 50 Pa at different temperatures (40, 50, 60 °C). 

#### 2.4.2. Scanning Electron Micriscope 

Scanning electron microscope (Hitachi S 4700, Tokyo, Japan) was used to observe the surface of morphology and the cross-section of the fibers at an accelerating voltage of 20 kV. The fibers were embedded with epoxy resin and fractured in the liquid nitrogen to observe the cross-section. The surface morphology of fibers were sputtered with white gold before SEM observation. 

#### 2.4.3. X-ray Diffraction

X-ray diffraction (Rigaku, Tokyo, Japan) patterns of the different fiber samples were measured on a D/max 2500VB2+/PC diffractometer using Cu Kα radiation between 5° and 55° at the rate of 10°/min and every 0.02°. The wavelength of the X-ray is 0.154056 nm. The degree of crystallization was calculated by the 2θ scan curves according to the fraction of integrated intensity of the crystalline zone around 2θ ≈ 16.8° to the total integrated intensity. The crystal size was estimated from the broadening of the main diffraction peak centered at 2θ ≈ 16.8° in the equatorial scan curves using the Scherrer equation with K = 0.89 [35]. Crystal region orientation was evaluated by the Herman orientation function. The azimuthal intensity was recorder by step scans at 0.2° intervals in azimuthal angle. 

#### 2.4.4. Fourier Transform Infrared Spectroscopy

Fourier transform infrared spectrum (Nicolet 5700 spectrometer, Thermo Nicolet Company, Madison, WI, USA) was recorded in the range of 450~4000 cm^−1^ at 4 cm^−1^ resolution using KBr pellets to observe the changes of chemical groups. Polarized FTIR was used to measure the orientation of nitrile groups for the PAN fibers depending on the intensity ratio of the parallel direction to the perpendicular direction for the peak at about 2243 cm^−1^. The end of fibers were fixed under slight tension, and the polarization beam was obtained depending on the different direction of incident FTIR beam.

#### 2.4.5. Raman Spectroscopy

The interfacial interaction between amino-CNTs and PAN macromolecular chains was characterized through Raman spectroscopy by using a Raman Confocal microscope (RM2000, Renishaw, UK) with a He–Ne laser (Spectra-Physics) excitation at 632.8 nm with a spot size 5 μm in diameter. The amino-CNTs were transferred onto Si wafers, and the composite fiber bundles were focused on the Si wafers along a certain direction. 

#### 2.4.6. Differential Scanning Calorimetry

Differential scanning calorimetry analysis was carried out by a Q 100 instrument (TA Company, Boston, PA, USA). The fibers with about 3.0 mg were heated from 50 to 400 °C at the heating rate 10 °C/min in nitrogen atmosphere. 

#### 2.4.7. Mechanical Properties

The mechanical properties of the precursor fibers and composite fibers were examined with a monofilament tensile testing machine produced by Nantong Hongda company (Jiangsu, China), and the speed rate is 5 mm/min at room temperature. Series of 25 single fiber were used for the measurements.

## 3. Results and Discussion

### 3.1. Rheological Behavior

**Figure 2:**
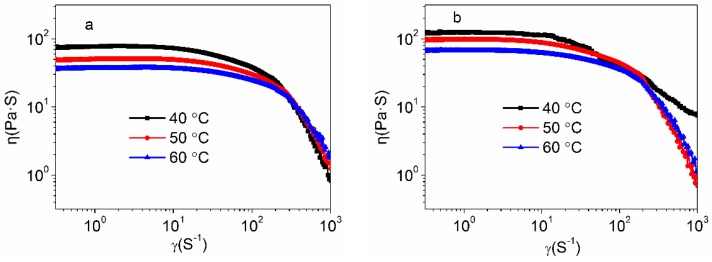
Complex viscosity of (**a**) the polyacrylonitrile (PAN) concentrated solutions and (**b**) the amino-functionalized multi-wall carbon nanotubes (amino-CNT)/PAN composite solutions at different temperatures.

The dependence of steady shear viscosities on the shear rate at different temperatures for the control PAN concentrated solutions and the amino-CNT/PAN composite concentrated solutions are shown in Figure 2. It is noted that all the samples exhibit shear thinning behavior. The control PAN concentrated solutions, as shown in Figure 2a, exhibit a typical Newtonian behavior at low shear rate region and shear thinning behavior at high shear rate region. However, the amino-CNT/PAN composite concentrated solutions, as shown in Figure 2b, show higher shear viscosity and earlier shear thinning behavior than the control PAN concentrated solution. The higher shear viscosity is assigned to the formation of the network structure in the composite concentrated solutions between the amino-CNTs and PAN macromolecular chains [32,36], which retarded the movement of the PAN chains at lower shear rate, leading to the higher viscosity. Therefore, the viscosity of the composite concentrated solutions are higher than that of the control PAN concentrated solutions at the same temperature. Moreover, the CNTs, a rigid one-dimension material, is easily oriented at higher shear rate, which facilitate disentanglement of PAN macromolecular chains. So, the amino-CNT/PAN concentrated solutions show earlier shear thinning behavior as compared to the control PAN concentrated solutions [29,37]. These behaviors are in agreement with the previous reports that the polymer filled with CNTs have similar shear thinning at relatively low shear rates [38]. These results also indicated that the amino-CNTs had good dispersion in the PAN concentrated solutions [39,40].

For the control PAN concentrated solutions and the amino-CNT/PAN composite concentrated solutions, the complex viscosity decreases with the increasing temperature, which can be explained by the free volume theory. The higher temperature allows more thermal motion of the PAN macromolecules chain and more free volume, which results in the decreasing of the intermolecular and intramolecular resistances associated with viscosity. At relative higher shear rates, the viscosity of samples improves with the increasing temperature, which is assigned to the sensitivity of viscosity to temperature less than that of the viscosity to shear rate. At 40 °C, the steady shear viscosity to shear rate of the amino-CNT/PAN composite concentrated solutions, as shown in Figure 2b, does not obviously descrease compared to the PAN solutions at high shear rate, which is assigned to the composite concentrated solutions less sensitive to the shear rate.

**Figure 3:**
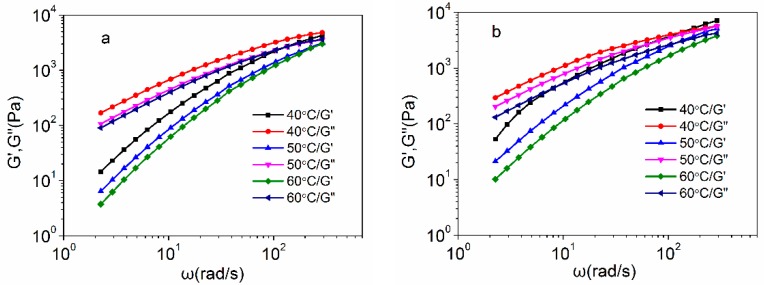
Storage modulus and loss modulus of (**a**) the PAN concentrated solutions and (**b**) the amino-CNT/PAN composite concentrated solutions at different temperatures.

Figure 3 shows the frequency dependences of the storage modulus (G’) and loss modulus (G”) for the control PAN concentrated solutions and amino-CNT/PAN composite concentrated solutions. It can be seen that both the value of G’ and G” generally increased with increasing frequency at the same temperature. The modulus significantly increased at low frequency region, and the increase of the G’ is larger than that of G”. The low frequency can be considered to reflect the relaxation and motion of the whole polymer chains. Therefore, the structure of the composite concentrated solutions is more sensitively reflected on the G’ than on the G”. At high frequency region, corresponding to the movement with small timescale, not many differences of the G’ with G” occur, which indicate that the movements of polymer chain segments are not affected by the addition of amino-CNTs. 

With temperature increasing, the value of G’ and G” reversely decrease, which is ascribed to the solutions having more free volume to provide the polymer chains moving. However, the value of G’ is lower than that of G” at low frequency for the same sample, which implies that these concentrated solutions show the liquid-like behavior. At high frequency in Figure 3b, especially for composite concentrated solutions at 40 °C, the value of G’ is higher than that of G”, suggesting that the transition from liquid-like to solid-like occurs. Chafidz et al. [32] reported that the polypropylene-nanoclay composites prepared from masterbatch had sol-to-gel transition point, and indicated that the polymer macromolecular could not completely relax owing to the interfacial interaction between the nanoclay and polymer matrix. Besides, the value of G’ for the amino-CNT/PAN composite concentrated solutions are higher than those of PAN concentrated solutions in low frequency at the same temperature, suggesting that the network structure could be formed between the amino-CNTs and PAN macromolecular chains [25,41,42]. Moreover, it is consistent with the uniform dispersion of amino-CNTs leading to the higher storage modulus [43,44]. 

**Figure 4:**
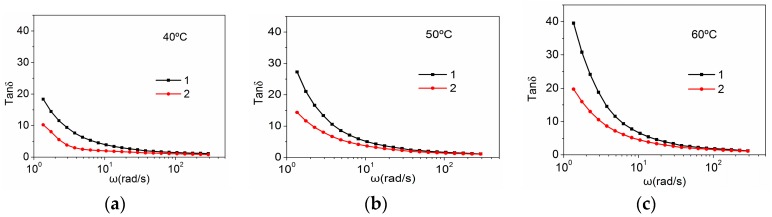
Tangent loss angle versus frequency of (1) the PAN concentrated solutions and (2) the amino-CNT/PAN composite concentrated solutions in the plot at different temperatures (**a**) 40 °C, (**b**) 50 °C and (**c**) 60 °C.

The relationships between the tangent loss angle (tanδ = G”/G’) and frequency at different temperatures are shown in Figure 4. With the temperature increasing, the value of tanδ increases in the low frequency. With the frequency increasing, the value of tanδ rapidly decreases at low frequency and tends to be stable at high frequency. Compared with the PAN concentrated solutions, the amino-CNT/PAN composite concentrated solutions at different temperatures, as shown in Figure 4, exhibit significantly lower tanδ at the lower frequency. The lower tanδ means that the materials show more solid-like behavior for the viscoelasticity. So, the addition of amino-CNTs improves the content of solid-like structure in the composite and decreases the liquid-like behavior at the low frequency, which are ascribed to forming the network structure between the amino-CNTs and the PAN macromolecular chains [45].

### 3.2. XRD Analysis

**Figure 5:**
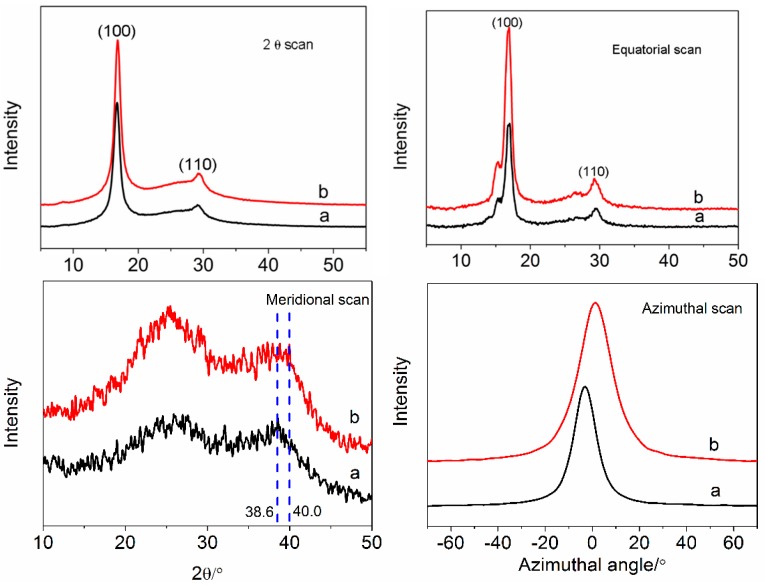
X-ray diffraction (XRD) curves of 2θ scan, equatorial scan, meridional scan and azimuthal scan for (a) PAN precursor fibers and (b) amino-CNT/PAN composite fibers.

The effects of amino-CNTs on the crystal structure and the orientation structure of PAN precursor fibers were investigated using XRD. 2θ scan of XRD for the fibers, as shown in Figure 5, was used to calculate the degree of crystallization. The PAN precursor fibers show a sharp and strong peak at about 16.8°, which is attributed to the (100) crystal plane of hexagonal lattice of PAN, and a weak peak at approximately 29.1° for the (110) crystal plane of PAN [13]. The amino-CNT/PAN composite fibers with higher relative intensity at about 16.8° show similar curves at the same positions having the diffraction peaks, which suggested that the addition of amino-CNTs into the PAN precursor fibers would not significantly change the crystal structure type during the processing of stretching. The equatorial scans of XRD were used to calculate the crystal size according to Scherrer equation [22]. The PAN precursor fibers and the amino-CNT/PAN composite fibers, as shown in Figure 5 (equatorial scan), have similar curves, too. But the intensity of peak at about 16.8° for the amino-CNT/PAN composite fibers is stronger than that of PAN precursor fibers. In the meridional scans, the peak at around 26° is assigned to the amorphous regions, and this peak of the amid-CNT/PAN composite fibers is significantly strong than that of the PAN precursor fibers, which is attributed to the (002) plane of CNTs in the composite fibers. The peaks at around 38.6° and 40.0° are ascribed to the planar zigzag conformation and helical conformation, respectively [21]. For the PAN precursor fibers, there is only one peak at 38.6°, indicating that the macromolecular chains arranged in the planar zigzag conformation. However, the amid-CNT/PAN composite fibers have two peaks in the conformation regions, and shows the peak of the helical conformation at about 40.0°. Kanamoto regarded that the PAN fibers with helical conformation had lower density than the PAN fibers with plane zigzag conformation owing to the smaller plane spacing [46], and this is consistent with density we tested in this paper. These results suggest that the addition of amid-CNTs into the PAN precursor fibers hindered the conformational changes from helical conformation to planar zigzag. The azimuthal scan was used to characterize the orientation of crystal region [22]. The amino-CNT/PAN composites fibers with larger full-width of half-maximum have relative high intensity compared with the PAN precursor fibers, suggesting that the addition of the amino-CNTs hindered the orientation of crystal region. 

The degree of crystallization, crystal size and crystal region orientation according to the different XRD curves are listed in Table 1. The PAN precursor fibers have higher degree of crystallization than the amino-PAN composite fibers. The macromolecular chains of PAN polymer would orientate and crystallize along the axis of the stretching force for preparing the PAN precursor fibers, which improve the degree of crystallization and crystal size compared to the nascent fibers. Owing to the interfacial interaction existing between amino-CNTs and PAN polymer, the amino-CNTs impede the motion and disrupt the ordered arrangement of PAN macromolecular chains during stretching processing. Mikolajczyk et al. [47] regarded that the lower degree of crystallization of composite fibers was assigned to the elimination of the crystal owing to the local concentration of stress. The composite fibers with lower crystal size than the PAN precursor fibers were attributed to the remaining crystallites orienting along the axis of the fiber. Therefore, the amino-CNT/PAN composite fibers have lower degree of crystallization and crystal size compared with the control PAN fibers. Moreover, the value of crystal orientation of composite fibers is lower than the PAN precursor fibers. 

### 3.3. FTIR Analysis

**Figure 6:**
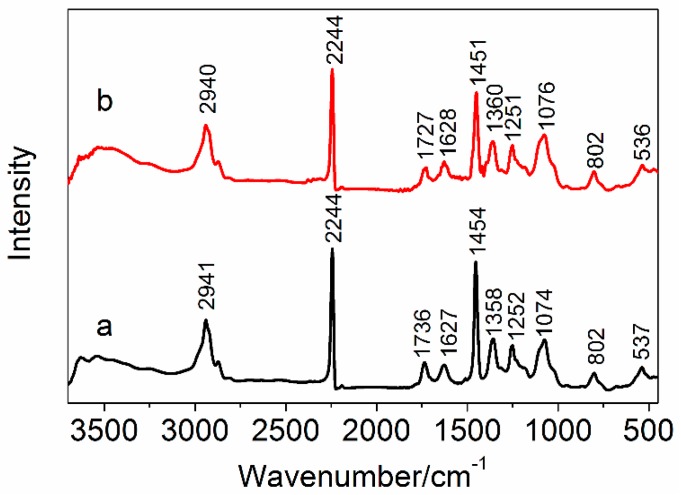
Fourier transform infrared (FTIR) spectra of (a) PAN precursor fibers and (b) amino-CNT/PAN composite fibers.

Figure 6 shows the chemical structure of the PAN precursor fibers and the amino-CNT/PAN composite fibers. The peaks of PAN precursor fibers in Figure 6a can be assigned as follows: 2941 cm^−1^ (–CH_2_ stretching), 2244 cm^−1^ (–C≡N stretching), 1736 cm^−1^ (–C=O stretching), 1627 cm^−1^ (–CONH_2_ stretching owing to hydrolysis of nitrile groups), 1454 cm^−1^ (–CH_2_ bending ), 1358 cm^−1^ (–CH bending), 1252 cm^−1^ (–CH deformation), 1074 cm^−1^ (–CN bending), 802 cm^−1^ (–CH wagging), 537 cm^−1^ (–C=O twisting) [48,49,50,51]. Some peaks of amino-CNT/PAN composite fibers shift compared to the PAN precursor fibers, especially for the peak at about 1736 cm^−1^. Sharifnejad et al. [48] reported that this peak of the carboxylic groups would shift to the higher wavenumbers with the content of second monomer (itaconic acid) increasing. According to the relative intensity of carboxylic groups and nitrile groups, the value of *I*_1736_/*I*_2244_ for the composite fibers is 0.135, which is lower than that of the PAN precursor fibers (0.152). These results correspond to the low contents of carboxylic groups with the low wavenumbers, indicating that the amino groups on the surface of CNTs take place as a chemical reaction with the carboxylic groups on the PAN macromolecular chains. The peak at about 1627 cm^−1^ is ascribed to the hydrolysis of nitrile groups. The ratio of *I*_1627_/*I*_2244_ of composite fibers is 0.152, which is higher than that of the PAN precursor fibers (0.122). These results suggest that the amino-CNTs have interfacial interaction with the nitrile groups on the PAN macromolecular chains and effect the processing of hydrolysis for the nitrile group. These shifts of some other peaks also mean that the amino-CNTs and PAN macromolecular chains have chemical interaction during the stretching. 

**Figure 7:**
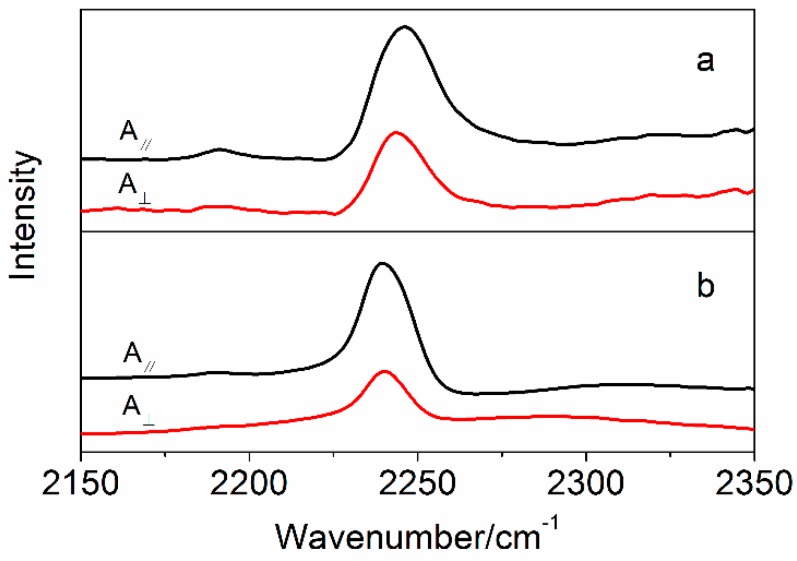
The polarized FTIR of (a) PAN precursor fibers and (b) amino-CNT/PAN composite fibers.

The polarized FTIR was used to characterize the orientation of nitrile groups in the PAN macromolecular chains [22]. Figure 7 shows the polarized FTIR of PAN precursor fibers and amino-CNT/PAN composite fibers. The peak at about 2244 cm^−1^ is ascribed to the nitrile groups, and the maximum of peak for the composite fibers have lower wavenumbers than that of the PAN precursor fibers. Eom et al. [52] regarded that the lower wavenumbers of nitrile groups were ascribed to the stronger dipole–dipole interaction between adjacent nitrile groups, and the helix conformation of PAN polymer induced the reorientation of nitrile groups. These results means that amino-CNT/PAN composite fibers with helix conformation have higher orientation of nitrile groups than the PAN precursor fibers. According to the relative intensity of nitrile groups, the ratio value of A_∥_/A_⊥_ for the PAN precursor fibers is 1.61, which is lower than that of the amino-CNTs/PAN composite fibers with 2.30. The higher orientation of nitrile groups in composite fibers is assigned to the amino-CNTs inducing the movement of nitrile groups owing to their strong interfacial interaction between them, and the CNTs have more easy orientation than the PAN macromolecular chains owing to having higher aspect ratio and rigid structure [53]. The results from the XRD meridional scans are in agreement with the results from the polarized FTIR analysis. 

### 3.4. Raman Spectroscopy

**Figure 8:**
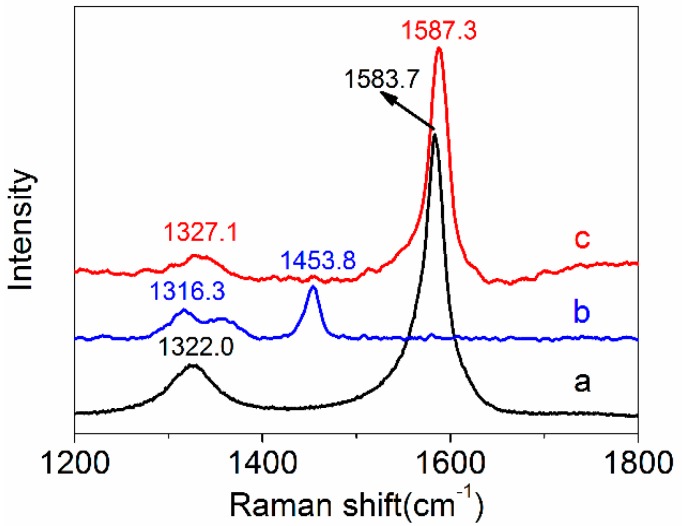
Raman spectra of (a) amino-CNTs, (b) PAN fibers, and (c) amino-CNT/PAN composite fibers.

Raman spectrum was used to characterize the interfacial interaction between the CNTs and polymer matrix according to the peak shift for the CNTs. Figure 8 shows the Raman spectra of the amino-CNTs, the PAN precursor fibers and the amino-CNT/PAN composite fibers. The amino-CNTs have two significant peaks. The strong peak at 1583.7 cm^−1^ is ascribed to the sp^2^ hybridized carbon (G-band), and the weak peak at 1322.0 cm^−1^ is assigned to the sp^3^ hybridized carbon (D-band) [54]. The PAN precursor fibers show two peaks at 1316.3 cm^−1^ and 1453.8 cm^−1^, which is ascribed to the –CH vibration peaks on the PAN macromolecular chains [55]. Although the mass content of amino-CNTs in the composite fibers is about 1%, the composite fibers exhibit two typical peaks of amino-CNTs. The first peak shift of amino-CNTs shifts from 1322.0 cm^−1^ to 1327.1 cm^−1^, and the second peak shifts from 1583.7 cm^−1^ to 1587.3 cm^−1^. The *R* value that is the ratio of the intensity of G-band to that of D-band was used to characterize the structural changes of CNTs. The value of *R* for the amino-CNTs is 5.53, which is higher than that of the composites fibers with 10.8. These results indicated that the surface of amino-CNTs in the composite fibers had more sp^2^ hybridized carbon after stretching. These results indicated that the amino-CNTs were de-bundled and homogeneously dispersed in the PAN fibers after stretching [26]. Meanwhile, the shift of G-band also indicated that the strong interfacial interaction existed between the amino-CNTs and the PAN macromolecular chains [13]. 

### 3.5. Morphology of Amino-CNT/PAN Composite Fiber

**Figure 9:**
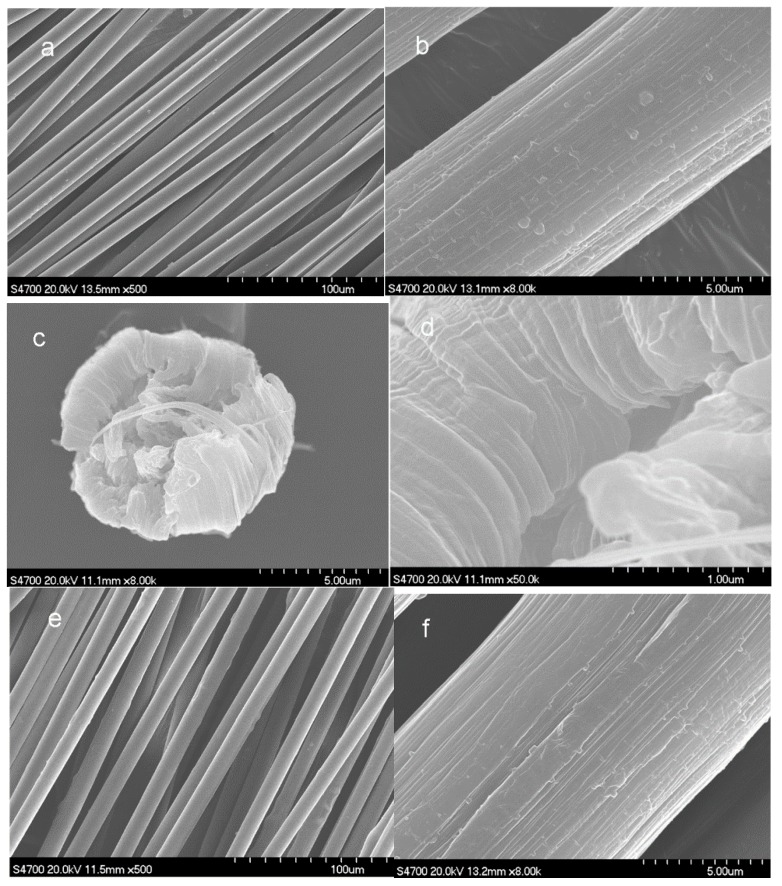

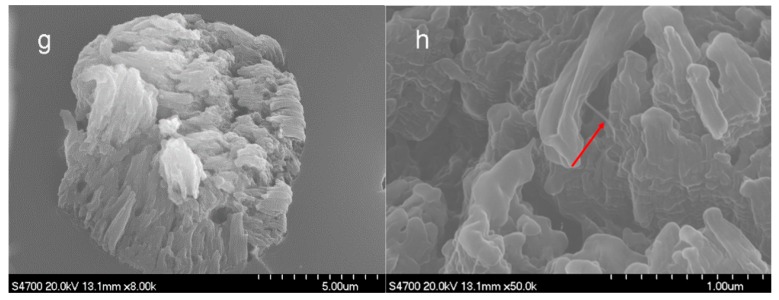
Scanning electron microscope (SEM) images of (**a**–**d**) the PAN precursor fibers and (**e**–**h**) the amino-CNT/PAN composite fibers: (**a**,**b**,**e**,**f**) the surface morphology of the fibers; (**c**,**d**,**g**,**h**) the cross-section of the fibers.

Figure 9 shows the SEM images of the PAN precursor fibers and the amino-CNT/PAN composite fibers. The surface morphology of PAN precursor fibers, as shown in Figure 9a, have some folded fragments arranging parallel to the oriented along the fiber axis direction. These structures are typical phenomena of PAN fibers obtained by wet-spinning after stretching. Similarly, the addition of amino-CNTs does not significantly change the surface morphology of PAN precursor fibers, as shown in Figure 9e. However, the order structure for the fibrous on the surface of amino-CNT/PAN composite fibers is not better than that of the PAN precursor fibers, which indicate that the incorporation of amino-CNTs into PAN polymer impeded the formation of the ordered structure for the PAN macromolecular chains after four-stage stretching. Additionally, the diameter of amino-CNT/PAN composite fibers is about 10.5 μm, which is larger than that of the PAN precursor fibers with about 8.0 μm. These are attributed to the composite concentrated solutions having higher viscosity than the control concentrated solutions, as confirmed by Figure 2, indicating that the PAN macromolecular chain has less time to relax through the spinneret hole. The cross-sections of the PAN precursor fibers and the amino-CNT/PAN composite fibers are nearly circular, as shown in Figure 9. The cross-section of PAN precursor fibers is similar to wavy grain, which is assigned to the crystallization and orientation of macromolecules along the axis. However, the morphology of composite fibers is different than that of the PAN precursor fibers, and the individual CNTs are connected between the fractured regions, as shown by the arrow in Figure 9h, which is in agreement with the forming network structure between the amino-CNTs and the PAN macromolecular chains. These results are also confirmed by rheological properties. 

### 3.6. DSC Curves

**Figure 10:**
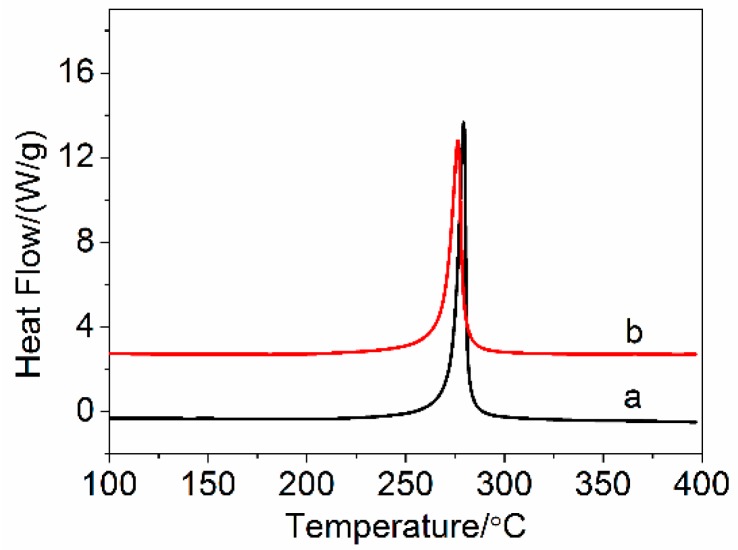
Differential scanning calorimetry (DSC) curves of (a) PAN precursor fibers and (b) amino-CNT/PAN composite fibers in nitrogen atmosphere.

Figure 10 shows the DSC curves of the PAN precursor fibers and the amino-CNT/PAN composite fibers under a nitrogen atmosphere at heating rate 10 °C/min. An intensive peak was exhibited in the temperature range 250~310 °C, which was attributed to the exothermic peak of cyclization reaction [33,56]. The amino-CNT/PAN composite fibers show lower intensive value and earlier maximum temperature of exothermic peak than the control PAN precursor fibers. The initial temperature (*T*_i_), the maximum temperature (*T*_m_), the finish temperature (*T*_f_), the evolved heat (Δ*H*), the difference between *T*_i_ and *T*_f_ (Δ*T*), and the velocity of evolving heat (Δ*H*/Δ*T*) are listed in Table 2. 

In Table 2, we can clearly see that the *T*_i_ of the amino-CNT/PAN composite fibers with 266.5° is significantly lower than that of the PAN precursor fibers with 258.3°, which can be attributed to the amino-CNTs inducing the onset of cyclization reaction [33,57]. The maximum temperature of the PAN precursor fibers shifts from 279.4 °C to 276.4 °C for the composite fibers, which is ascribed to the composite fibers having lower degree of crystallization than the PAN precursor fibers, and this is also confirmed by the XRD analysis. The finish temperature for both samples are not basically changed. Moreover, the amino-CNT/PAN composite fibers have larger exothermic temperature range and lower evolved heat than the PAN precursor fibers, which make the composite fibers having more modest velocity of evolving heat during cyclization than the PAN precursor fibers. These results indicated that the cyclization of the amino-CNT/PAN composite fibers is easier controlled than the PAN precursor fibers during the processing of thermal stabilization. 

### 3.7. Mechanical Properties of Amino-CNT/PAN Composite Fibers

The density and mechanical properties of the PAN precursor fibers and the amino-CNT/PAN composite fibers are listed in Table 3. The CNT/PAN composite fibers have lower density than the PAN precursor fibers, which is ascribed to the composite fibers having lower degree of crystallization and helical conformation. However, the incorporation of CNT into the PAN precursor fibers with larger diameter enhances the tensile strength from 0.74 ± 0.06 to 0.83 ± 0.05 GPa and the tensile modulus from 16.03 ± 1.01 to 28.37 ± 1.70 GPa, and decreases the breaking elongation from 10.73 ± 0.86 to 8.79 ± 0.53%. These results are consistent with the previous literature [18]. Meanwhile, the higher strength of composite fibers with lower degree of crystallization suggested that the crystallization structure was not the main factor to influence the mechanical properties owing to the amino-CNTs [58]. 

## 4. Conclusions

In this paper, the effects of amino-CNTs on the rheological behavior of PAN concentrated solutions prepared by in situ polymerization were studied. The addition of amino-CNTs into the PAN concentrated solutions with 22% concentration by mass have the higher shear viscosity and show earlier shear thinning behavior compared to the control PAN concentrated solutions, indicating that the network structures were formed between the amino-CNTs and the PAN macromolecular chains and the amino-CNTs could be homogeneously dispersed in the PAN concentrated solutions. The amino-CNT/PAN composite concentrated solutions with higher storage modulus at low frequency suggested that there existed network structures and the uniform dispersion of amino-CNTs in the composite concentrated solutions, which were also confirmed by the amino-CNT/PAN composite concentrated solutions having lower tangent loss angle at the same conditions. The composite fibers containing 1% amino-CNT by mass have 46.5% degree of crystallization, 77.7 Å crystal size and 0.914 orientation of crystal region, which is lower than the control PAN precursor fibers with 50.5% degree of crystallization, 83.4 Å crystal size and 0.943 orientation of crystal region. These results coming from the XRD analysis suggested that the addition of amino-CNTs hindered the orientation and crystallization of the PAN chains during the processing of stretching. The results from FTIR and Raman spectroscopy indicated that the amino-CNTs had interfacial interaction with the PAN macromolecular chains, and affected the orientation of nitrile groups, which made that the ratio value of A_∥_/A_⊥_ for the control PAN precursor fibers increased from 1.61 to 2.30 for the amino-CNT/PAN composite fibers. The SEM images for the amino-CNT/PAN composite fibers prepared by wet-spinning method exhibit the diameter of about 10.5 μm, which is larger than the control PAN precursor fibers with diameter of about 8.0 μm. This is ascribed to the composite solutions having higher viscosity than the PAN concentrated solutions. The initial temperature of cyclization reaction for composite fibers is 258.3 °C, which is significantly lower than that of the control PAN precursor fibers with 266.5 °C. The addition of amino-CNTs into the PAN precursor fibers also decreases the maximum temperature from 279.4 °C to 276.4 °C. The cyclization of composite fibers have larger exothermic temperature range and lower evolved heat than the PAN precursor fibers. Furthermore, the tensile strength and tensile modulus of the amino-CNT/PAN composite fibers are 0.83 GPa and 28.37 GPa, respectively, which is higher than the control PAN precursor fibers with the tensile strength of 0.74 GPa and the tensile modulus of 16.03 GPa.

## Figures and Tables

**Table 1:** X-ray diffraction (XRD) data of the polyacrylonitrile (PAN) precursor fibers and the amino-functionalized multi-wall carbon nanotubes (amino-CNT)/PAN composite fibers.

Samples	Degree of Crystallization (%)	Crystal Size (Å)	Orientation of Crystal Region
PAN	50.5	83.4	0.943
Amino-CNT/PAN	46.5	77.7	0.914

**Table 2:** Differential scanning calorimetry (DSC) data of the PAN precursor fibers and the amino-CNT/PAN composite fibers in nitrogen atmosphere.

Samples	*T*_i_ (°C)	*T*_m_ (°C)	*T*_f_ (°C)	Δ*H* (J/g)	Δ*T* (°C)	Δ*H*/Δ*T* (J/(g·°C))
PAN	266.5	279.4	285.7	489.8	19.2	25.51
Amino-CNT/PAN	258.3	276.4	285.8	473.7	27.5	17.23

**Table 3:** The mechanical properties of the PAN precursor fibers and the amino-CNT/PAN composite fibers.

Samples	Density (g/cm^3^)	Tensile Strength (GPa)	Tensile Modulus (GPa)	Breaking Elongation (%)
PAN	1.185 ± 0.002	0.74 ± 0.06	16.03 ± 1.01	10.73 ± 0.86
Amino-CNT/PAN	1.164 ± 0.002	0.83 ± 0.05	28.37 ± 1.70	8.79 ± 0.53

## References

[1] Levshov D., Than T.X., Arenal R., Popov V.N., Parret R., Paillet M., Jourdain V., Zahab A.A., Michel T., Yuzyuk Y.I. (2011). Experimental evidence of a mechanical coupling between layers in an individual double-walled carbon nanotube. Nano Lett..

[2] Song B.J., Ahn J.W., Cho K.K., Roh J.S., Lee D.Y., Yang Y.S., Lee J.B., Hwang D.Y., Kim H.S. (2013). Electrical and mechanical properties as a processing condition in polyvinylchloride multi walled carbon nanotube composites. J. Nanosci. Nanotechnol..

[3] Souier T., Santos S., AI Ghaferi A., Stefancich M., Chiesa M. (2012). Enhanced electrical properties of vertically aligned carbon nanotube-epoxy nanocomposites with high packing density. Nanoscale Res. Lett..

[4] Kueseng P., Sae-oui P., Rattanasom N. (2013). Mechanical and electrical properties of natural rubber and nitrile rubber blends filled with multi-wall carbon nanotube: Effect of preparation methods. Polym. Test..

[5] Malik S., Krasheninnikov A.V., Marchesan S. (2018). Advances in nanocarbon composite materials. Beilstein J. Nanotechnol..

[6] Gulotty R., Castellino M., Jagdale P., Tagliaferro A., Balandin A.A. (2013). Effects of functionalization on thermal properties of single-wall and multi-wall carbon nanotube-polymer nanocomposites. ACS Nano.

[7] Szatkowski P., Pielichowska K., Blazewicz S. (2017). Mechanical and thermal properties of carbon nanotube reinforced self-healing polyurethanes. J. Mater. Sci..

[8] Marchesan S., Melchionna M., Prato M. (2015). Wire up to carbon nanostructures! How to play a winning game. ACS Nano.

[9] Melchionna M., Prato M. (2013). Functionalizing carbon nanotubes: An indispensible step towards applications carbon nanostructures and devices. ECS J. Solid State Sci. Technol..

[10] Bai Y., Zhang Y., Wang Q., Wang T. (2013). Shape memory properties of multi-walled carbon nanotube/polyurethane composites prepared by in situ polymerization. J. Mater. Sci..

[11] Zhang K., Lim J.Y., Choi H.J., Lee J.H., Choi W.J. (2013). Ultrasonically prepared polystyrene/multi-walled carbon nanotube nanocomposites. J. Mater. Sci..

[12] Saeed K., Park S.Y., Haider S., Baek J.B. (2009). In situ polymerization of multi-walled carbon nanotube/nylon-6 nanocomposites and their electrospun nanofibers. Nanoscale Res. Lett..

[13] Zhang H., Xu L., Yang F., Geng L. (2010). The synthesis of polyacrylonitrile/carbon nanotube microspheres by aqueous deposition polymerization under ultrasonication. Carbon.

[14] Casey J.P., Bachilo S.M., Moran C.H., Weisman R.B. (2008). Chirality-resolved length analysis of single-walled carbon nanotube samples through shear-aligned photoluminescence anisotropy. ACS Nano.

[15] Barman S.N., LeMieux M.C., Baek J., Rivera R., Bao Z. (2010). Effects of dispersion conditions of single-walled carbon nanotubes on the electrical characteristics of thin film network transistors. ACS Appl. Mater. Interfaces.

[16] Xiao H., Lu Y., Zhao W., Qin X. (2014). A comparison of the effect of hot stretching on microstructures and properties of polyacrylonitrile and rayon-based carbon fibers. J. Mater. Sci..

[17] Wang W., Murthy N.S., Chae H.G., Kumar S. (2008). Structural changes during deformation in carbon nanotube-reinforced polyacrylonitrile fibers. Polymer.

[18] Chae H.G., Sreekumar T.V., Uchida T., Kumar S. (2005). A comparison of reinforcement efficiency of various types of carbon nanotubes in polyacrylonitrile fiber. Polymer.

[19] Sreekumar T.V., Chandra L., Srivastava A., Kumar S. (2007). Oxidative stabilization of polyacrylonitrile in the presence of functionalized carbon nanotubes. Carbon.

[20] Newcomb B.A., Chae H.G., Gulgunje P.V., Gupta K., Liu Y., Tsentalovich D.E., Pasquali M., Kuamr S. (2014). Stress transfer in polyacrylonitrile/carbon nanotube composite fibers. Polymer.

[21] Li X., Ji X., Qin A., He C. (2015). The plasticized spinning and cyclization behaviors of functionalized carbon nanotube/polyacrylonitrile fibers. RSC Adv..

[22] Quan L., Zhang H., Xu L. (2015). Orientation and thermal properties of carbon nanotube/polyacrylonitrile nascent composite fibers. J. Polym. Res..

[23] Ray S.S., Okamoto M. (2003). Polymer/layered silicate nanocomposites: A review from preparation to processing. Prog. Polym. Sci..

[24] Akhlaghi O., Akbulut O., Menceloglu Y.Z. (2015). Shear and extensional rheological characterization of poly(acrylonitrile)/halloysite nanocomposite solutions. Eur. Polym. J..

[25] Martins J.N., Kersch M., Altstädt V., Oliveira R.V.B. (2013). Poly(vinylidene fluoride)/polyaniline/carbon nanotubes nanocomposites: Influence of preparation method and oscillatory shear on morphology and electrical conductivity. Polym. Test..

[26] Gupta A., Choudhary V. (2013). Rheologic and mechanical properties of multiwalled carbon nanotubes-reinforced poly(trimethylene terephthalalte) composites. J. Mater. Sci..

[27] Zonder L., Ophir A., Kenig S., McCarthy S. (2011). The effect of carbon nanotubes on the rheology and electrical resistivity of polyamide 12/high density polyethylene blends. Polymer.

[28] Chae D.W., Hong S.M. (2011). Rheology, crystallization behavior under shear, and resultant morphology of PVDF/multiwalled carbon nanotube composites. Macromol. Res..

[29] Lalko M.P., Rakesh L., Hirschi S. (2009). Rheology of polycarbonate reinforced with functionalized and unfunctionalized single-walled carbon nanotubes. J. Therm. Anal. Calorim..

[30] Amr I.T., Al-Amer A., Thomas S., Al-Harthi M., Girei S.A., Sougrat R., Atieh M.A. (2011). Effect of acid treated carbon nanotubes on mechanical, rheological and thermal properties of polystyrene nanocomposites. Compos. Part B Eng..

[31] Yan X.L., Gong Z.L., Gong J., Gao S., Zhang Z., Wang B. (2012). Investigation of the rheological and conductive properties of multi-walled carbon nanotube/polycarbonate composites by positron annihilation techniques. Carbon.

[32] Kim K.S., Park S.J. (2011). Influence of amine-grafted multi-walled carbon nanotubes on physical and rheological properties of PMMA-based nanocomposites. J. Solid State Chem..

[33] Zhang H., Quan L., Xu L. (2017). Effects of amino-functionalized carbon nanotubes on the crystal structure and thermal properties of polyacrylonitrile homopolymer microspheres. Polymers.

[34] Quan L., Zhang H., Xu L. (2015). The non-isothermal cyclization kinetics of amino-functionalized carbon nanotubes/polyacrylonitrile composites by in situ polymerization. J. Therm. Anal. Calorim..

[35] Bashir Z. (1992). Thermoreversible gelation and plasticization of polyacrylonitrile. Polymer.

[36] Chafidz A., Ali M.A., Elleithy R. (2011). Morphological, thermal rheological, and mechanical properties of polypropylene-nanoclay composites prepared from masterbatch in a twin screw extruder. J. Mater. Sci..

[37] Akhlaghi O., Menceloglu Y.Z., Akbulut O. (2016). Rheological behavior of poly(acrylonitrile) concentrated solutions: Effect of Sb_2_O_3_ nanoparticles on shear and extensional flow. Colloid Polym. Sci..

[38] Xu D.H., Wang Z.G. (2008). Influence of carbon nanotube aspect ratio on normal stress differences in isotactic polypropylene nanocomposite melts. Macromolecules.

[39] Zhang K., Choi H.J. (2015). Fabrication and viscoelastic characteristics of amino-functionalized multi-walled carbon nanotube/poly(methyl methacrylate) nanocomposites. Compos. Struct..

[40] Mitchell C.A., Bahr J.L., Arepalli S., Tour J.M., Krishnamoori R. (2002). Dispersion of functionalized carbon nanotubes in polystyrene. Macromolecular.

[41] Ke K., Wang Y., Yang W., Xie B.H., Yang M.B. (2012). Crystallization and reinforcement of poly(vinylidene fluoride) nanocomposites: Role of high molecular weight resin and carbon nanotubes. Polym. Test..

[42] Kim K.S., Byun J.H., Lee G.H., Park S.J. (2011). Influence of GMA grafted MWNTs on physical and rheological properties of PMMA-based nanocomposites by in situ polymerization. Macromol. Res..

[43] Bose S., Bhattacharyya A.R., Kulkarni A.R., Pötschke P. (2009). Electrical, rheological and morphological studies in co-continuous blends of polyamide 6 and acrylonitrile–butadiene–styrene with multiwall carbon nanotubes prepared by melt blending. Compos. Sci. Technol..

[44] Xu Z., Niu Y., Yang L., Xie W., Li H., Gan Z., Wang Z. (2010). Morphology, rheology and crystallization behavior of polylactide composites prepared through addition of five-armed star polylactide grafted multiwalled carbon nanotubes. Polymer.

[45] Ke K., Wen R., Wang Y., Yang W., Xie B.H., Yang M.B. (2011). Crystallization behavior of poly(vinylidence fluoride)/multi-walled carbon nanotubes nanocomposites. J. Mater. Sci..

[46] Sawai D., Yamane A., Kameda T., Kanamoto T. (1999). Uniaxial drawing of isotactic poly(acrylonitrile): Development of oriented structure and tensile properties. Macromolecules.

[47] Mikolajczyk T., Rabiej S., Szparaga G., Bogun M., Fraczek-Szczypta A., Blazewicz S. (2009). Strength properties of polyacrylonitrile (PAN) fibres modified with carbon nanotubes with respect to their porous and supramolecular structure. Fibres Text. East Eur..

[48] Sharifnejad F., Bahrami S.H., Noorpanah P. (2005). Kinetics studies on copolymerization of acrylonitrile vinyl acids by solvent-water suspension polymerization. J. Appl. Polym. Sci..

[49] Santhana Krishnan G., Thomas P., Murali N. (2016). Synthesis, characterization, and thermo-mechanical properties of poly(acrylonitrile-*co*-2,3-dimethyl-1,3-butadiene-*co*-itaconic acid) as carbon fibre polymer precursors. RSC Adv..

[50] Simitzis J.C., Georgiou P.C. (2015). Functional group changes of polyacrylonitrile fibres during their oxidative, carbonization and electrochemical treatment. J. Mater. Sci..

[51] Karacan I., Erdogan G. (2012). The influence of thermal stabilization stage on the molecular structure of polyacrylonitrile fibers prior to the carbonization stage. Fiber Polym..

[52] Eom Y., Park Y., Jung Y.M., Kim B.C. (2017). Effects of conformational change of polyacrylonitrile on the aging behavior of the solutions in *N*,*N*-dimethyl formamide. Polymer.

[53] Cai J., Chawla S., Naraghi M. (2016). Microstructural evolution and mechanics of hot-drawn CNT-reinforced polymeric nanofibers. Carbon.

[54] Ma H.Y., Tong L.F., Xu Z.B., Fang Z.P. (2008). Functionalizing carbon nanotubes by grafting on intumscent flame retardant: Nanocomposite synthesis, morphology, rheology, and flammability. Adv. Funct. Mater..

[55] Edwards H.G.M., Hoskins A.R., Johnson A.F., Lewis I.R. (1993). Raman spectroscopic studies of the polyacrylonitrile-zinc complexes in aqueous solutions of zinc chloride and bromide. Polym. Int..

[56] Bajaj P., Sreekumar T.V., Sen K. (2001). Thermal behavior of acrylonitrile copolymers having methacrylic and itaconic acid comonomers. Polymer.

[57] Zhou T., Wang X., Wang T. (2009). Cure reaction of multi-walled carbon nanotubes/diglycidyl ether of bisphenol A/2-ethyl-4-methylimidazole (MWCNTs/DGEBA/EMI-2,4) nanocomposites: Effect of carboxylic functionalization of MWCNTS. Polym. Int..

[58] Zhou H., Tang X., Dong Y., Chen L., Zhang L., Wang W., Xiong X. (2011). Multiwalled carbon nanotube/polyacrylonitrile composite fibers prepared by in situ polymerization. J. Appl. Polym. Sci..

